# Plasma sRAGE enables prediction of acute lung injury after cardiac surgery in children

**DOI:** 10.1186/cc11354

**Published:** 2012-05-22

**Authors:** XiWang Liu, QiXing Chen, ShanShan Shi, Zhuo Shi, Ru Lin, LinHua Tan, JianGen Yu, Qiang Shu, XiangMing Fang

**Affiliations:** 1 Department of Thoracic & Cardiovascular Surgery, Children's Hospital, Medical College, Zhejiang University, and Key Laboratory of Reproductive Genetics (Zhejiang University), Ministry of Education, Hangzhou 310003, China; 2 Department of Anesthesiology, the First Affiliated Hospital, Medical College, Zhejiang University, Hangzhou 310003, China

## Abstract

**Introduction:**

Acute lung injury (ALI) after cardiac surgery is associated with a high postoperative morbidity and mortality, but few predictors are known for the occurrence of the complication. This study evaluated whether elevated plasma levels of soluble receptor for advanced glycation end products (sRAGE) and S100A12 reflected impaired lung function in infants and young children after cardiac surgery necessitating cardiopulmonary bypass (CPB).

**Methods:**

Consecutive children younger than 3 years after cardiac surgery were prospectively enrolled and assigned to ALI and non-ALI groups, according to the American-European Consensus Criteria. Plasma concentrations of sRAGE and S100A12 were measured at baseline, before, and immediately after CPB, as well as 1 hour, 12 hours, and 24 hours after operation.

**Results:**

Fifty-eight patients were enrolled and 16 (27.6%) developed postoperative ALI. Plasma sRAGE and S100A12 levels increased immediately after CPB and remained significantly higher in the ALI group even 24 hour after operation (*P *< 0.01). In addition, a one-way MANOVA revealed that the overall sRAGE and S100A12 levels were higher in the ALI group than in the non-ALI group immediately after CPB (*P *< 0.001). The multivariate logistic regression analysis showed that the plasma sRAGE level immediately after CPB was an independent predictor for postoperative ALI (OR, 1.088; 95% CI, 1.011 to 1.171; *P *= 0.025). Increased sRAGE and S100A12 levels immediately after CPB were significantly correlated with a lower PaO_2_/FiO_2 _ratio (*P *< 0.01) and higher radiographic lung-injury score (*P *< 0.01), as well as longer mechanical ventilation time (sRAGE_N_: *r *= 0.405; *P *= 0.002; S100A12_N_: *r *= 0.322; *P *= 0.014), longer surgical intensive care unit stay (sRAGE_N_: *r *= 0.421; *P *= 0.001; S100A12_N_: *r *= 0.365; *P *= 0.005) and hospital stay (sRAGE_N_: *r *= 0.329; *P *= 0.012; S100A12_N_: *r *= 0.471; *P *= 0.001).

**Conclusions:**

Elevated sRAGE and S100A12 levels correlate with impaired lung function, and sRAGE is a useful early biomarker of ALI in infants and young children undergoing cardiac surgery.

## Introduction

Postoperative lung injury may occur in 12% to 50% of patients undergoing cardiac surgery necessitating cardiopulmonary bypass (CPB) [1-4], and up to 20% of the patients need ventilation for more than 48 hours [5]. Acute respiratory distress syndrome (ARDS) occurs in 2% of these cases, resulting a mortality of 15% to 50% [3,4]. Children are more prone to acute lung injury (ALI) under the detrimental stimulation during cardiac surgery with CPB [6-8]. ALI/ARDS after CPB often leads to a prolonged length of hospital stay and increased therapeutic costs. Therefore, developing certain predictive biomarker for CPB-related ALI/ARDS in infants and young children undergoing cardiac surgery would be of great help for early diagnosis and efficient therapeutic decision making.

The receptor for advanced glycation end products (RAGE) is a member of the immunoglobulin superfamily that acts as a multiligand receptor and progression factor in propagating the inflammatory response [9,10]. RAGE is expressed at a high level in the basal surface of alveolar type I cells, and its expression is upregulated under inflammatory conditions [9,11,12]. Soluble RAGE (sRAGE) is the extracellular form of RAGE and is produced by either cleaving the membrane by proteolysis or removing the transmembrane region via alternative splicing [12]. Increasing evidence suggests that local and circulating levels of sRAGE are promising as biomarkers of pulmonary tissue damage. The sRAGE concentration has been reported to be dramatically higher in pulmonary edema fluid and plasma from adult patients with ALI/ARDS, which were caused by sepsis, trauma, or primary pneumonia [13,14]. Furthermore, increased plasma sRAGE levels are significantly correlated with the severity and clinical outcome of ALI/ARDS [15,16].

S100A12 is a member of the S100 family of calcium-binding proteins, which is a newly identified extracellular prototypic ligand of RAGE [17,18]. S100A12 expression may reflect neutrophil activation and contribute to pulmonary inflammation and endothelial activation via binding to RAGE [14,18]. Blocking the interaction of S100A12 and RAGE results in improvement of inflammation in multiple experimental models [18,19]. Wittkowski *et al. *[14] found that compared with healthy controls, patients with ARDS caused by pneumonia or peritonitis had significantly enhanced S100A12 expression in pulmonary tissue and higher S100A12 concentrations in bronchoalveolar lavage fluid.

Plasma sRAGE was recently reported to be a sensitive and rapid marker of lung distress after elective coronary artery bypass grafting in a study of 20 adult patients [20]. Additionally, Kikkawa and colleagues [21] found that sepsis patients who developed postoperative ALI had higher S100A12 levels compared with controls. Initial studies are promising. However, it is not clear whether these findings could extend to younger children undergoing cardiac surgery with CPB.

This pilot study aimed to observe the kinetics of plasma sRAGE and S100A12 in infants and young children undergoing cardiac surgery with CPB and to investigate whether plasma sRAGE and S100A12 levels are associated with the occurrence and severity of ALI after cardiac surgery.

## Materials and methods

### Study population

This prospective study was conducted at a university children's hospital located in eastern China. The study protocols were approved by the hospital ethics committee (Medical Ethical Committee of the Children's Hospital of Zhejiang University), and informed consents were signed by supervisors of the patients. Children who were younger than 3 years and scheduled for cardiac surgery for congenital heart disease (CHD) were consecutively enrolled. The included patients had stable clinical conditions for at least 2 months. Patients were excluded if they were premature, had abnormal liver or renal function, had major chromosomal abnormalities, showed pulmonary inflammation before the surgery, had pulmonary edema due to cardiac dysfunction, needed extracorporeal membrane oxygenation support after the operation, died because of cardiac dysfunction, or refused to participate in the study.

### Data collection and definitions

During the surgical procedure, all patients underwent routine hemodynamic and blood gas surveillance. Anesthesia, the CPB procedure, and weaning from mechanical ventilation (MV) in the surgical Intensive Care Unit (ICU) were all performed by using standard protocols, as shown in additional material in detail [Additional file 1].

Demographic and preoperative data were collected, including the patient's gender, age, weight, pulse oximetry saturation (SpO_2_), Risk Adjusted Classification for Congenital Heart Surgery (RACHS-1), ultrafiltrate volume, CPB time and operation time, duration of MV, and the ratio of fraction of inspired oxygen to oxygen pressure (PaO_2_/FiO_2_). A bedside chest radiograph was taken every day after surgery. A structured tutorial was used to establish consensus in the interpretation of radiographs for radiographic lung-injury scores (LISs) [16,22]. Echocardiography was performed routinely to evaluate the cardiac function after surgery and at any time if necessary. The left ventricular function was evaluated by the ejection fraction (EF). The North American-European Consensus Criteria were used to categorize the patients into the ALI group and non-ALI group. In brief, this criterion includes the acute onset of bilateral alveolar infiltrates seen on a chest radiograph, a PaO_2_/FiO_2 _ratio < 300, and the absence of cardiogenic pulmonary edema (CPE) [23]. CPE was identified when the pulmonary arterial occlusion pressure was > 18 mm Hg or by the presence of at least two of the following: central venous pressure > 14 mm Hg, left ventricular EF < 45%, systemic hypertension, or volume overload. Patients with CPE were excluded; CPE was diagnosed by two independent attending cardiac intensivists, who were blinded to the group of ALI patients. In addition, the both groups were followed up for ICU length of stay (LOS) and hospital LOS.

### Determination of sRAGE and S100A12 in plasma

For each patient, 2 ml of fresh blood was drawn into a vacuum tube containing EDTA at the following time points: before operation, before CPB, after CPB, 1 hour, 12 hours, and 24 hours after operation. After being centrifuged at 3,000 rpm for 15 minutes at 4°C, the plasma was divided into aliquots and frozen at -80°C until assay.

sRAGE and S100A12 levels were measured by using the commercially available ELISA kits (sRAGE, R&D Systems, Minneapolis, MN, USA; S100A12, Cirulex; Cyclex Co. Ltd, Nagano, Japan), according to the manufacturer's instructions. The plasma total protein levels were determined with an autobiochemistry analyzer and used for sRAGE and S100A12 normalization (sRAGE_N_, sRAGE normalized for total protein; S100A12_N_, S100A12 normalized for total protein). Laboratory staffs were blinded to the ALI patients, and investigators involved in the interpretation of ALI were blinded to sRAGE and S100A12 levels.

### Statistical analysis

Continuous data were tested for normal distribution with the one-sample Kolmogorov-Smirnov test. Variables were presented as mean values and standard deviations if normally distributed, and otherwise, as median values (interquartile range). The Student *t *test and Mann-Whitney *U *test were used to determine the significance of variable differences between the two groups. The χ^2 ^test or Fisher Exact test were used to compare categorical data as appropriate. A multivariate analysis of variance procedure (MANOVA) was used to assess whether a significant difference remained in the overall assessment of sRAGE and S100A12 levels between the two groups. A Pearson or Spearman correlation test was performed to determine the correlation between biomarker (sRAGE and S100A12) data and clinical parameters (CPB time, PaO_2_/FiO_2 _ratio, MV time, and ICU and hospital LOS). Receiver operating characteristic curve (ROC) was computed, and area under the curve (AUC) was used to evaluate how well the biomarkers diagnose ALI. A stepwise logistic regression model was used to determine the independent risk factor for ALI after CPB. The multivariate variables included age, weight, sex, CPB time, operation time, and the concentration of sRAGE_N _and S100A12_N _immediately after CPB. These variables were then entered into a stepwise multiple linear regression analysis to determine the factors significantly associated with the PaO_2_/FiO_2 _ratio within the first 2 days. A *P *value < 0.05 was considered statistically significant. All statistical analyses were performed by using SPSS (SPSS 16.0 for Windows; SPSS, Chicago, IL, USA).

## Results

### Study population

From May 1 to June 30, 2011, of the 71 consecutive patients younger than 3 years who underwent cardiac surgery, 58 (81.7%) fulfilled the inclusion criteria, which included 27 (46.6%) infants. Thirteen patients were excluded for the following reasons: pneumonia before surgery (eight patients), liver insufficiency (one patient), prematurity (two patients), receiving cardiopulmonary resuscitation owing to low-cardiac-output syndrome (one patient), and absence of written informed consent (one patient). Finally, among the 58 patients, 16 (27.6%) developed ALI. The characteristics and cardiac-lesion types of the enrolled patients are shown in Tables 1 and 2, respectively.

**Table 1:** Demographic and clinical characteristics of the patients enrolled in the study cohort

	Study cohort (*n *= 58)	ALI (*n *= 16)	Non-ALI (*n *= 42)	*P *value
Sex male *n *(%)	28 (48.3%)	10 (62.5%)	18 (42.9%)	0.181
Age (months)	12.2 ± 5.8	6.7 ± 4.0	14.3 ± 5.0	< 0.001
Weight (kg)	8 ± 2	6.3 ± 1.9	8.6 ± 1.6	< 0.001
RACHS-1				0.409
≤2	49	12	37	
≥3	9	4	5	
SPO_2 _(%)	97 (95-98)	95 (90-97)	98 (96-98)	0.002
Operation time (minutes)	131.9 ± 30.4	148.7 ± 20.4	125.4 ± 31.3	0.008
CPB time (minutes)	65.5 ± 22.8	81.1 ± 15.82	59.5 ± 22.4	0.001
UFV (ml)	328.1 ± 128.2	365.6 ± 142.4	313.81 ± 121.1	0.171
Nosocomial pneumonias	9 (15.5%)	4 (25%)	5 (11.9%)	0.409
MV time (hours)	7 (4.8-26.2)	27 (26.2-34.4)	5.2 (4.3-11.8)	< 0.001
SICU LOS (days)	4.44 ± 2.8	7.6 ± 2.9	3.4 ± 1.6	< 0.001
Hospital LOS (days)	20 ± 7.8	26.8 ± 9.4	17.4 ± 5.2	< 0.001

Data are presented as number of patients (%), median (interquartile range), or counts, as appropriate. ALI, acute lung injury; CPB, cardiopulmonary bypass; LOS, length of stay; MV, mechanical ventilation; RACHS-1, Risk Adjusted Classification for Congenital Heart Surgery; SICU, surgery intensive care unit; SPO_2_, oxygen saturation; UFV, ultrafiltered volume removal.

**Table 2:** Cardiac lesion types in the study cohort

Type of lesion	Study cohort	ALI	Non-ALI
VSD plus ASD	13	3 (18.8)	10 (23.8)
VSD	14	2 (12.5)	12 (28.6)
ASD	4	0 (0)	4 (9.5)
TOF	6	2 (12.5)	4 (9.5)
TAPVC plus ASD or VSD	5	2 (12.5)	3 (7.1)
Atrioventricular canal defect	7	3 (18.8)	4 (9.5)
DORV plus PS	4	2 (12.5)	2 (4.8)
PA plus VSD plus PDA	5	2 (12.5)	3 (7.1)
Total	58	16	42

Data are presented as counts (%). ASD, atrial septal defect; DORV, double-outlet right ventricle; PS, pulmonary stenosis; TAPVC, total anomalous pulmonary venous connection; TOF, tetralogy of Fallot; VSD, ventricular septal defect.

### Perioperative sRAGE and S100A12 concentrations

**Figure 1:**
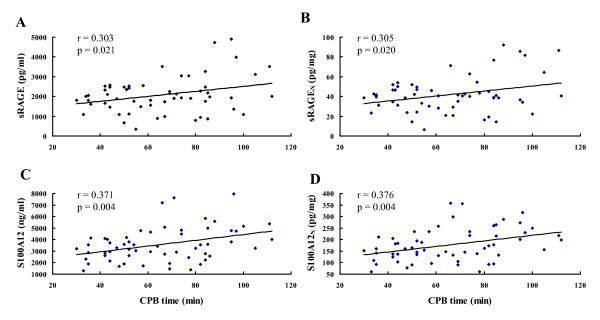
**Scatterplot displaying the relation between CPB time and plasma sRAGE and S100A12 levels immediately after CPB**. CPB, cardiopulmonary bypass.

Plasma sRAGE and S100A12 concentrations are shown in Table 3, both as absolute values (picograms per milliliter) and normalized values (picograms per milligram total protein). The latter values were needed because total plasma protein concentration decreased dramatically during CPB (*P *< 0.001). Plasma levels of sRAGE and S100A12 increased significantly immediately after CPB (*P *< 0.001), and the elevated levels were correlated with the CPB time (Figure 1; sRAGE: *r *= 0.303; *P *= 0.021; sRAGE_N_: *r *= 0.305; *P *= 0.02; S100A12: *r *= 0.371; *P *= 0.004; S100A12_N_: *r *= 0.376; *P *= 0.004). Therefore, the normalized values (sRAGE_N _and S100A12_N_, respectively) immediately after CPB were used for subsequent analysis. Twenty-four hours after operation, the levels of sRAGE decreased lower than the baseline levels (*P *< 0.05), whereas S100A12 levels remained higher (*P *< 0.001).

**Table 3:** Perioperative gas-exchange parameters and plasma sRAGE and S100A12 data

Variables	Before surgery	Before CPB	After CPB	1 hour after surgery	12 hours after surgery	24 hours after surgery
PaO_2 _(mm Hg)		191.5 ± 60.5	185.3 ± 77.2	156.2 ± 40.4	146.2 ± 39.3	111.4 ± 20.8
PaCO_2 _(mm Hg)		34.4 ± 4.5	40.5 ± 7.4	36.6 ± 5.7	37.1 ± 4.6	37.2 ± 5.2
FiO_2_	Room air	0.47 ± 0.9	0.60 ± 0.19	0.50 ± 0.04	0.45 ± 0.1	0.33 ± 0.15
Total protein (g/L)	63.74 ± 4.76	51.99 ± 4.05	49.76 ± 4.78^a^	51 ± 4.35^b^	55.4 ± 5.47^c^	55.18 ± 6.35^d, e^
sRAGE (pg/ml)	821.28 ± 267.93	995.11 ± 568.81	2,073.91 ± 911.82^a^	1,587.15 ± 657.35^b, f^	753.74 ± 460.27^c, g^	508.38 ± 270.03^d, e^
sRAGE_N _(pg/mg)	12.91 ± 4.3	20 ± 12.12	41.8 ± 18.25^a^	31.52 ± 13.75^b, f ^	13.87 ± 8.81^g^	9.4 ± 5.3^d, e ^
S100A12 (ng/ml)	16.32 ± 11.92	22.89 ± 15.20	176.69 ± 70.53^a^	193.04 ± 62.56^b^	198.13 ± 71.45^c^	176.19 ± 78.43^d^
S100A12_N _(pg/mg)	255.3 ± 185.8	458.49 ± 298.74	3,570.99 ± 1,473.74^a^	3,805.61 ± 1,296.3^b^	3,593.58 ± 1,309.83^c ^	3,474.96 ± 1,254.31^d^

Data are presented as mean ± SD, unless otherwise noted. PaO_2_, arterial oxygen tension; PaCO_2_, arterial carbon dioxide tension; FiO_2_, inspiratory oxygen fraction; sRAGE_N_, sRAGE normalized for total protein amount; S100A12_N_, S100A12 normalized for total protein amount. ^a^*P *< 0.001 after CPB versus before surgery; ^b^*P *< 0.001 1 hour after surgery versus before surgery; ^c^*P *≤ 0.05 12 hours after surgery versus before surgery; ^d^*P *≤ 0.002 24 hours after surgery versus before surgery; ^e^*P *< 0.001 24 hours after surgery versus after CPB; ^f^*P *= 0.001 1 hour after surgery versus after CPB; ^g^*P *< 0 .001 12 hours after surgery versus after CPB.

### Plasma sRAGE as a predictor for acute lung injury

**Figure 2:**
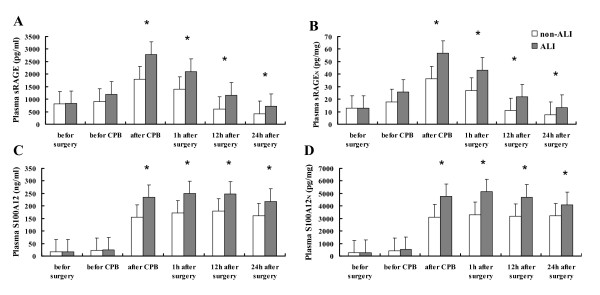
**Perioperative courses over time of plasma sRAGE and S100A12 levels in the acute lung injury (ALI) and non-ALI groups**. Data are expressed as mean ± standard error of the mean. *P < 0.01.

The post-CPB ALI development not only was associated with age, weight, operation time, and CPB time (*P *< 0.01) (Table 1), but also was significantly associated with the levels of sRAGE and S100A12 immediately after CPB (Figure 2; *P *< 0.01). In addition, the levels of sRAGE and S100A12 were kept higher in the ALI group than those in non-ALI group at 24 hours after operation (Figure 2; *P *< 0.01). The one-way MANOVA analysis revealed that ALI patients had significantly increased overall biomarkers of sRAGE and S100A12, as compared with the non-ALI ones, both immediately after CPB and 24 hours after operation (*F *= 29.06; *P *< 0.001 and *F *= 11.72; *P *< 0.001, respectively).

**Figure 3:**
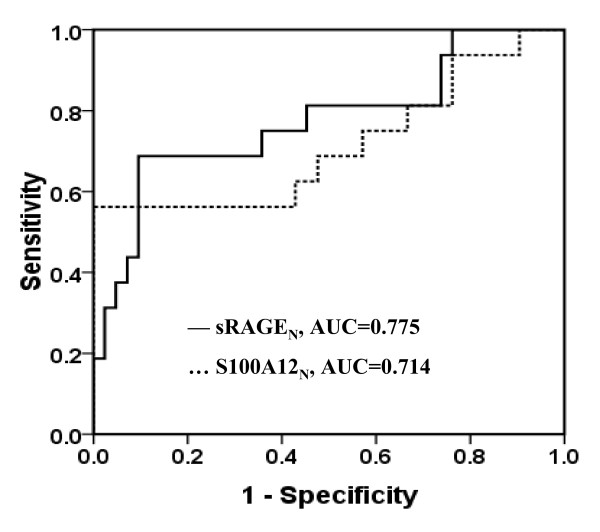
**Receiver operating characteristic curves displaying the ability of plasma levels of sRAGE_N _and S100A12_N _to predict the occurrence of ALI**. The AUCs of sRAGE_N _and S100A12_N _for ALI were 0.775 (95% CI, 0.626 to 0.924) and 0.714 (95% CI, 0.539 to 0.889), respectively. ALI, acute lung injury; AUC, area under the curve.

In the ROC analysis, the AUC of sRAGE_N _and S100A12_N _levels immediately after CPB for ALI were 0.775 (95% CI, 0.626 to 0.924) and 0.714 (95% CI, 0.539 to 0.889), respectively. At a cutoff value of 54 pg/mg, sRAGE_N _had a sensitivity of 70% and a specificity of 91% for diagnosis of ALI after CPB, whereas at a cutoff of 4,326.5 pg/mg, S100A12_N _had a sensitivity of 58% and a specificity of 100% (Figure 3). The logistic regression analysis, which included age, weight, sex, operation time, and CPB time, as well as S100A12_N _and sRAGE_N _levels immediately after CPB, revealed that the independent risk factors for the occurrence of ALI were sRAGE_N _(OR, 1.088; 95% CI, 1.011 to 1.171; *P *= 0.025), age (OR, 0.681; 95% CI, 0.528 to 0.879; *P *= 0.003), and CPB time (OR, 1.070; 95% CI, 1.008 to 1.136; *P *= 0.026).

### Plasma sRAGE and S100A12 predict the severity of acute lung injury and clinical outcomes

**Figure 4:**
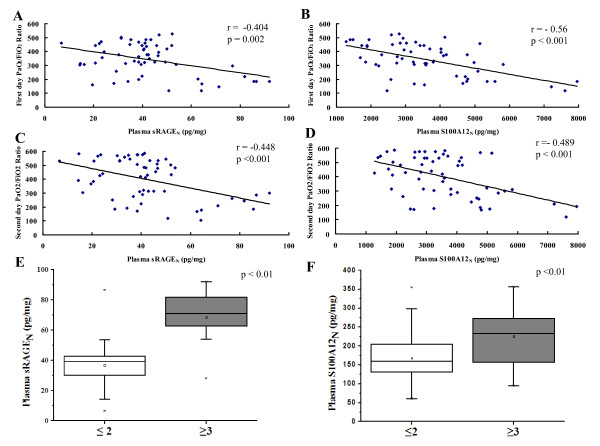
**Plasma sRAGE_N _and S100A12_N _levels immediately after CPB reflecting the PaO_2_/FiO_2 _ratio (A-D) and radiographic LIS (E, F)**. LIS, lung-injury score.

Higher levels of plasma sRAGE_N _and S100A12_N _immediately after CPB were significantly associated with more-severe ALI, as reflected by the measurement of pulmonary physiology, including the PaO_2_/FiO_2 _ratio on the first 2 days (Figure 4A and 4B for the first day; sRAGE_N_: *r *= -0.404; *P *= 0.002; S100A12_N_: *r *= -0.56; *P *< 0.001; Figure 4C and 4D for the second day: sRAGE_N_: *r *= -0.448; *P *< 0.001; S100A12_N_: *r *= -0.489; *P *< 0.001), as well as radiographic LIS (Figure 4E and 4F; *P *< 0.01). After adjusting for age, weight, sex, operation time, and CPB time in a multiple linear regression model, we found that sRAGE_N _but not S100A12_N _levels immediately after CPB were independently associated with the PaO_2_/FiO_2 _ratio on the second day (B = -2.153; 95% CI, -0.559 to -3.747; *P *= 0.009); however, none of them was associated with the ratio within 24 hours after surgery.

**Figure 5:**
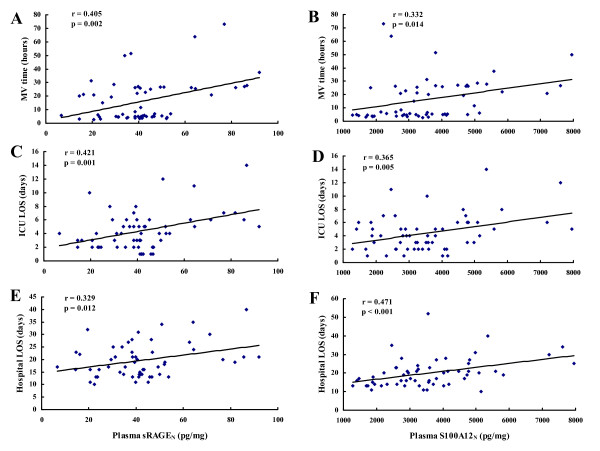
**Scatterplot displaying the relation between plasma sRAGE_N _and S100A12_N _levels immediately after CPB and the clinical outcomes**. The mechanical ventilation time **(A, B)**, the ICU LOS **(C, D)**, and the hospital LOS **(E, F) **are shown.

Likewise, elevated sRAGE_N _and S100A12_N _levels were correlated with longer MV time (Figure 5A and 5B; sRAGE_N_: *r *= 0.405; *P *= 0.002; S100A12_N_: *r *= 0.322; *P *= 0.014), surgical ICU LOS (Figure 5C and 5D; sRAGE_N_: *r *= 0.421; *P *= 0.001; S100A12_N_: *r *= 0.365; *P *= 0.005) and hospital LOS (Figure 5E and 5F; sRAGE_N_: *r *= 0.329; *P *= 0.012; S100A12_N_: *r *= 0.471; *P *= 0.001).

## Discussion

In this prospective pilot study of the kinetics of plasma sRAGE and S100A12 levels in infants and young children undergoing cardiac surgery with CPB, we found that plasma concentrations of sRAGE and S100A12 remarkably increased immediately after CPB. The elevated levels were correlated with the severity of ALI and clinical outcomes of cardiac surgery with CPB and plasma sRAGE was a reliable early predictor for the occurrence of ALI after cardiac surgery.

Uniquely, RAGE is expressed at remarkably high basal levels in lung tissues [9,11,12], suggesting that it holds important functions in both pulmonary physiology and numerous pathologic states [9,12]. The normal values of sRAGE were reported to range from 500 to 1,250 pg/ml in healthy volunteers [20,21,24,25]. In addition, no significant difference was found in normal plasma sRAGE levels among various ages and in baseline levels with respect to the cause of lung injury, such as aspiration pneumonia, and to coexisting conditions, such as diabetes, end-stage renal disease, essential hypertension, or other vascular diseases [13]. Previous studies provided supportive evidence that the primary source of plasma sRAGE in ALI/ARDS patients was alveolar type I cells [11-13,24], and sRAGE served as a marker of alveolar epithelial injury [15,16,24]. As expected, in the current study, we found that the plasma levels of sRAGE were significantly higher in the ALI group than in the non-ALI group immediately after CPB. After adjusting the ALI-related variants, sRAGE remained as an independent risk factor for ALI development. Furthermore, we found the sRAGE levels were related with the impaired lung function, such as lower PaO_2_/FiO_2 _ratio and higher radiographic LIS. These findings suggested that plasma sRAGE levels may serve as a reliable quantitative predictor for the development of post-CPB ALI in infants and young children. Because plasma was diluted during CPB, the normalized plasma sRAGE and S100A12 concentrations were used to define the cut-off values (sRAGE, 54 pg/mg; S100A12, 4,326.5 pg/mg) in our study. Nevertheless, the cut-off value should be used with caution to diagnose ALI in a clinical setting, because the defined abnormal sRAGE or S100A12 levels were unequal among different studies in different patients [20,21,24,25]. Unfortunately, to date, only a few studies exist on sRAGE and S100A12, which were always based on small number of patients. Hence, a larger-cohort validation study is needed to verify these results in future.

Previous studies have shown that the elevated circulation levels of sRAGE after onset of ALI/ARDS soon decreased to lower levels [13,14,17,18]. Interestingly, we found similar kinetics of plasma sRAGE during CPB in children, to those in adult patients receiving coronary artery bypass grafting [20]. One possible explanation for this decrement is the reoxygenation resulting from reperfusion of the lung after CPB, because Lizotte *et al. *[26] reported that hyperoxic exposures lead to a loss of sRAGE in the neonatal rat lung. In addition, a great number of activated neutrophils during extracorporeal circulation may explain the decreasing sRAGE levels [27]. sRAGE is known to have multiple protease-sensitive sites, so it might be degraded by neutrophil-derived proteolytic enzymes [28].

Infiltration of activated neutrophils is an important hallmark of ALI [29]; therefore more neutrophils in ALI may account for the high levels of S100A12 in the ALI group. Although neutrophils and their secretory products play important roles in the pulmonary inflammation, such as ALI, they are a rather unspecific surrogate for lung-tissue injury [29-31], which may contribute to the disassociation of plasma levels of S100A12 with the occurrence and the severity of post-CPB ALI in the logistic analysis and the multiple linear regression analysis.

In addition, the present study found that both plasma levels of sRAGE and S100A12 immediately after CPB were positively correlated with the duration of MV, surgical ICU LOS, and hospital LOS, and that sRAGE showed an independent association with the PaO_2_/FiO_2 _ratio on the second day. Although speculative, the following reasons might explain these correlations. In ALI/ARDS patients, Mauri *et al. *[15] reported that the first-day plasma sRAGE levels were correlated with the release of CRP and pentraxin 3 (PTX3) in the circulation [15], which promoted the coagulation/fibrinolysis dysfunction and organ failures [15,32]. RAGE interacts with leukocyte Mac-1 integrin, monocyte chemoattractant protein-1, and CD11c/CD18 to facilitate inflammatory cell recruitment [33,34]. Attraction of leukocytes to an inflammation site is additionally augmented by interaction with RAGE ligands such as S100A12 [10,14,18,32]. Furthermore, interaction of RAGE and S100A12 causes production of proinflammatory cytokines including TNF-α, IL-6, and IL-8 [12,21], which induced increased lung cell damage and vascular permeability [34,35]. Taken together, RAGE and S100A12 contribute to more severe and prolonged inflammation in the lung and damage of the alveolar capillary barrier with increased lung water content and impaired oxygenation, resulting in longer MV support and more critical care in the SICU after cardiac surgery. There was no doubt that the S100A12-RAGE axis played an important role in the development of ALI after cardiac surgery with CPB. Because several single nuclear polymorphisms of the RAGE gene were recently found to affect the expression of sRAGE and associate with lung function in pathologic states [36-38], it should be emphasized that genetic variation of RAGE must be considered in further studies.

Limitations of the present study should be acknowledged. Although the kinetics of plasma sRAGE levels in this study is consistent with that in a published study [20], our findings were based on a small number of patients in a single center, which might limit the application of these findings to other institutions. Furthermore, we enrolled the patients undergoing surgery for CHD in the absence of relevant comorbidities and particularly of known lung disease. Therefore, we are not sure whether the sRAGE and S100A12 changes would be the same in the presence of previous lung diseases. Because children often have pneumonias before cardiac operations, future studies must confirm this.

Interestingly, Jabaudon *et al. *[13] reported that sRAGE levels in ALI/ARDS were not influenced by the presence of RAGE-related diseases such as sepsis, diabetes mellitus, end-stage renal disease, coronary artery disease, rheumatoid arthritis, Alzheimer disease, and essential hypertension.

Third, the traditional inflammatory cytokines were not measured in this study. However, none of them was a specific biomarker for post-CPB ALI, although they were found to change perioperatively. In addition, *in vitro *study showed that the induction of S100A12 was more prominent than other cytokines, such as IL-8 [14], suggesting that the S100A12-RAGE axis may be more important in the CPB-induced inflammatory response.

## Conclusions

We found that plasma sRAGE acts as a reliable early biomarker of ALI after cardiac surgery with CPB in infants and young children. The plasma levels of sRAGE and S100A12 are associated with lung function and clinical outcomes after CPB. These data may indicate that RAGE is a mediator of lung injury in patients after cardiac surgery and not merely a maker of disease. Insight into the role of the ligand-RAGE axis in the post-cardiac surgery inflammatory response holds the potential for better understanding of ALI induced by CPB.

## Key messages

• Early identification of the occurrence of acute lung injury (ALI) would facilitate efficient therapeutic decision making in infants and young children after cardiac surgery with cardiopulmonary bypass (CPB).

• Plasma sRAGE and S100A12 levels increased after CPB, and the plasma levels of sRAGE immediately after CPB showed high predictive values for the development of ALI in infants and young children after cardiac surgery necessitating CPB.

• Increased sRAGE and S100A12 levels immediately after CPB significantly reflected the severity of ALI and are correlated with longer mechanical ventilation time, surgical ICU length of stay, and hospital length of stay.

• Further studies are required to confirm the value of sRAGE in predicting ALI after cardiac surgery with CPB in a larger population.

## Abbreviations

ALI: acute lung injury; ARDS: acute respiratory distress syndrome; CHD: congenital heart disease; CPB: cardiopulmonary bypass; MV: mechanical ventilation; LIS: lung-injury score; LOS: length of stay; sRAGE: soluble receptor for advanced glycation end products; RACHS-1: risk adjustment for surgery for congenital heart disease.

## Competing interests

The authors declare that they have no competing interests.

## Authors' contributions

XWL, QXC, QS, and XMF contributed to study conception and design, data analysis and interpretation, drafting of the manuscript, and critical revision of the article for important content. XWL, SSS, and ZS contributed to data collection, analysis, and interpretation and to drafting of the manuscript. XWL, QXC, SSS, ZS, RL, LHT, JGY, QS, and XMF contributed to data analysis and interpretation, drafting of the manuscript, and critical revision of the article for important content. All authors read and approved the manuscript for publication.

## Supplementary Material

Additional file 1 **Standard protocols for anesthesia, cardiopulmonary bypass, and weaning from mechanical ventilation**. Additional file 1 is the standard protocols for anesthesia, cardiopulmonary bypass, and weaning from mechanical ventilation, which were used in all patients. Click here for file
